# Supplementary material for: ALDOB is a prognostic biomarker and a potential immunotherapy target for clear cell renal cell carcinoma
Source: PeerJ. 2025 Aug 18;13:e19869. doi: 10.7717/peerj.19869 (PMC12369605; doi:10.7717/peerj.19869)
Supplement: Supplemental Information 2 [file peerj-13-19869-s002.docx]

**Original author and contributions**

**Wu Xu**: Writing-review & editing, Writing-original draft, **Yufeng Liu**: Visualization, Software, **Cuilian Li** and **Bo Peng**: Methodology, Investigation, **Yang Luo**: Data curation, Conceptualization. **Dawei Liu**: Writing-original draft, Software, **Lingfei Yan**: Methodology, Investigation, Data curation. **Qing Li**: Writing-review & editing, Validation, **Tao Wang**: Funding acquisition, Conceptualization.

**Change to**

**Wu Xu**: Writing-review & editing, Writing-original draft, **Dali Wu** : Visualization, Software, **Cuilian Li** and **Bo Peng**: Methodology, Investigation, **Yang Luo**: Data curation, Conceptualization. **Dawei Liu**: Writing-original draft, Software, **Lingfei Yan**: Methodology, Investigation, Data curation. **Qing Li**: Writing-review & editing, Validation, **Tao Wang**: Funding acquisition, Conceptualization.

**New author and contributions**

Dali Wu : Visualization, Software. Used bioinformatics software and other online tools to obtain the results for Figures 1-4 and Tables 1-2, and also participated in the image layout work.
